# Reduced Root Cortical Tissue with an Increased Root Xylem Investment Is Associated with High Wheat Yields in Central China

**DOI:** 10.3390/plants13081075

**Published:** 2024-04-11

**Authors:** Pengzhen Du, Yong-He Zhu, Jacob Weiner, Zhengli Sun, Huiquan Li, Tao Feng, Feng-Min Li

**Affiliations:** 1 School of Architecture and Urban Planning, Lanzhou Jiaotong University, Lanzhou 730070, China; dupz@lzu.edu.cn; 2 Jiangsu Collaborative Innovation Center for Modern Crop Production, College of Agriculture, Nanjing Agricultural University, Nanjing 210095, China; muzhen2008@outlook.com; 3 Department of Plant and Environmental Sciences, University of Copenhagen, DK-1871 Frederiksberg, Denmark; jw@plen.ku.dk; 4 State Key Laboratory of Grassland Agroecosystems, Institute of Arid Agroecology, School of Ecology, Lanzhou University, Lanzhou 730000, China; sunzhl21@lzu.edu.cn (Z.S.); hqli@lzu.edu.cn (H.L.); 5 Key Laboratory of Eco-Environment-Related Polymer Materials of Ministry of Education, Key Laboratory of Polymer Materials of Gansu Province, College of Chemistry and Chemical Engineering, Northwest Normal University, Lanzhou 730070, China

**Keywords:** anatomical traits, carbon allocation, cortex area, population yield, *Triticum aestivum*, water transport, winter wheat, xylem area

## Abstract

Trait-based approaches are increasingly used to understand crop yield improvement, although they have not been widely applied to anatomical traits. Little is known about the relationships between root and leaf anatomy and yield in wheat. We selected 20 genotypes that have been widely planted in Luoyang, in the major wheat-producing area of China, to explore these relationships. A field study was performed to measure the yields and yield components of the genotypes. Root and leaf samples were collected at anthesis to measure the anatomical traits relevant to carbon allocation and water transport. Yield was negatively correlated with cross-sectional root cortex area, indicating that reduced root cortical tissue and therefore reduced carbon investment have contributed to yield improvement in this region. Yield was positively correlated with root xylem area, suggesting that a higher water transport capacity has also contributed to increased yields in this study. The area of the leaf veins did not significantly correlate with yield, showing that the high-yield genotypes did not have larger veins, but they may have had a conservative water use strategy, with tight regulation of water loss from the leaves. This study demonstrates that breeding for higher yields in this region has changed wheat’s anatomical traits, reducing the roots’ cortical tissue and increasing the roots’ xylem investment.

## 1. Introduction

Researchers are attempting to apply ecological and evolutionary approaches to enhancing crop population yield [1]. A growing number of ecologists suggest adopting trait-based approaches to understand crop improvements [2]. Breeding for increased yields under agricultural conditions has altered plants’ anatomical, as well as other more frequently studied, traits [3].

**Figure 1:**
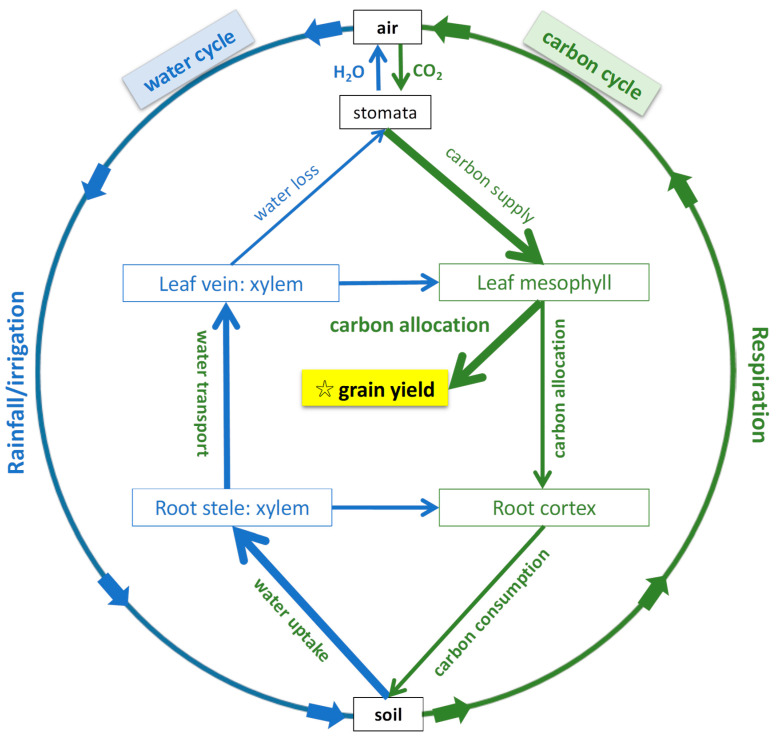
Framework of the water cycle (blue lines) and carbon cycle (green lines) in crops. Crops uptake water from the soil through their root hairs and then transport it through apoplastic or symplastic/transmembrane roots to the xylem vascular system. Water transported through stem moves through the xylem via vessels and then distinct orders of veins in leaves to the bundle sheath and mesophyll tissue, before being transpired into the air through stomata. The assimilates produced by photosynthesis in leaves are transported in the opposite direction to the spikes (yield formation), stems and roots via the phloem. More carbon being allocated to grains and less allocated downward to living root cortex for respiration can increase yield. Root and leaf anatomy play important roles in water and carbon cycles and yield production [13,14].

Anatomical traits could help explain crops’ individual ecological functions, such as carbon allocation and water transport (Figure 1), and, in turn, illuminate some mechanisms of population yield improvement [3]. The anatomical traits of the roots, such as their cross-sectional cortex, stele and xylem areas, reflect their radial and axial carbon and water use [4,5], influencing growth and yield in crops [6]. It has been hypothesized that reduced living cortical tissue in the roots with fewer cortical cells and increased root cortical aerenchyma is associated with lower root respiration and reduced metabolic costs, enabling deeper soil water acquisition [7,8,9,10,11]. In most cases, a reduced root cortical investment can also reduce resistance to water transport from the soil to the xylem, which is beneficial for radial water transport and absorption [12]. Thus, roots’ cortical traits have multiple functions related to carbon consumption and water transport [6]. In addition, a large stele can improve hydraulic conductance and axial water transport efficiency [10]. Variations in the ratio of the cortex to the stele can reflect trade-offs between carbon consumption and water transport in the roots [12].

Breeding for increased yields has resulted in smaller, deeper and more vertical root systems, which have increased the population yield by reducing the competition among wheat plants for water close to the surface and increasing access to water deeper in the soil under many agricultural conditions [15,16]. It has been hypothesized that a reduced living cortex burden and/or increased root cortical aerenchyma reduce(s) metabolic maintenance and construction costs, allowing the roots to explore deeper water and improving drought tolerance and yield under drought and low-phosphorus conditions in maize [7,11,14]. Evolutionary agricultural theory predicts a trade-off between individual fitness and population yield among productive genotypes [17]. A reduced cortical investment with relatively small, vertical roots may represent a strategy of decreased individual competitive capacity and increased communal behavior to increase the population yield [16]. Simultaneously, a reduced xylem area may reduce water use during flowering, increasing water use efficiency. A previous study concluded that wheat genotypes with low axial conductance conserved water during grain filling, resulting in higher yields under drought [18]. Following this approach, we predict that winter wheat has been selected due to its reduced root cortical tissue for deep water acquisition and reduced xylem and stele tissue for conservative water use efficiency.

Leaf anatomy also plays important roles in carbon and water management. Mesophyll tissues in the leaves produce carbon assimilates via photosynthesis, some of which are remobilized to the reproductive organs for the formation of grains and spikes or partitioned downward to the stems and roots (Figure 1). It is well documented that increasing reproductive allocation (Harvest Index or reproductive allometry) has been a major contributor to increased yields [19,20,21,22,23,24]. It is reasonable to hypothesize that a larger leaf mesophyll area will result in increased carbon assimilation and less carbon being partitioned to the stem and root tissues; some of this carbon may be allocated to increased reproduction (yield). In addition, the number of leaf veins plays a critical role in regulating water loss in wheat. “Ideotypes” with a higher grain yield generally have compact and short phenotypes with a conservative water use strategy and a high water use efficiency [25]. We hypothesize that higher-yield genotypes will have increased leaf mesophyll tissue and a reduced leaf vein investment to optimize the leaves’ assimilation capability (increase source capacity) per unit of leaf area and reduce transpiration, thereby increasing their water use efficiency. Thus, a reduced leaf vein investment, together with reduced root xylem tissue, may contribute to water use efficiency for the whole plant and promote high yields.

Here, we investigate the anatomical root and leaf traits at anthesis of 20 winter wheat genotypes grown in Luoyang, located in the Huanghuai Plain of China, which is the major region of wheat production in China, playing a critical role in national food [26]. We explore the relationships between root and leaf anatomical traits and yield and hypothesize that higher yields are associated with (1) a reduced root cortical investment, (2) a reduced root water transport capacity and (3) increased leaf mesophyll tissue, reflecting the attributes of an “ideotype” under local conditions.

## 2. Materials and Methods

### 2.1. Plant Materials and Growth Conditions

The field study was performed in Luoyang County, Henan Province, in central China, which had an average annual precipitation of 650 mm and an average annual temperature of 13 °C from 1979 to 2018, according to the China Meteorological Forcing Dataset [27]. We investigated 20 genotypes of winter wheat that were bred at or near the site and are commonly grown in the region (Table 1). Some of the genotypes, such as Bainong207 and Zhoumai32, have a high drought tolerance and a high disease tolerance.

**Figure 2:**
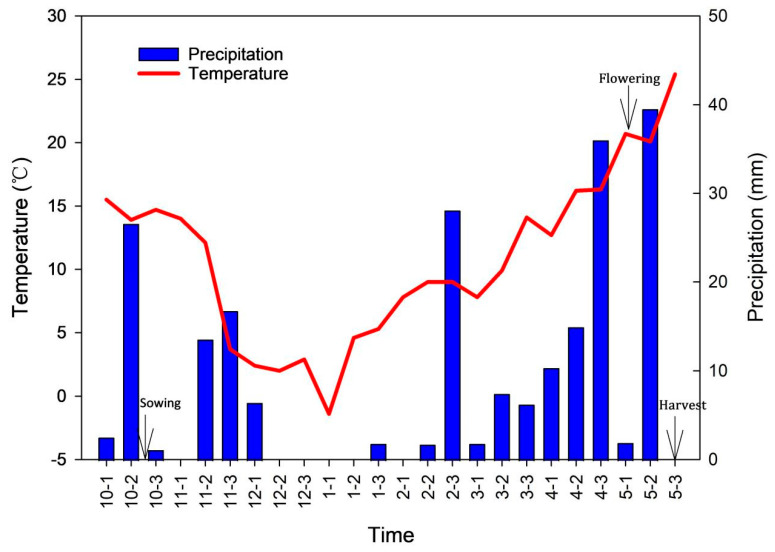
Average temperature and the sum of precipitation over 10-day periods during the growth of winter wheat from October to May in Luoyang, Henan Province. *x*-axis: 10−1 refers to the first 10 days of October, 10−2 to the middle 10 days of October and 10−3 to the remainder of October.

A field experiment was conducted from October 2020 to May 2021 using a standard local planting density (300 seeds per m^2^) to compare the yields. The average temperature and the total precipitation over 10-day periods during the growing season were recorded by the local weather station (Figure 2). The lowest temperature in winter was below −5 °C in 2020 (Figure 2), and the daily temperature increased gradually during the spring. There was little rainfall in December 2020 and January 2021 (Figure 2).

The field study was a randomized block design with three replicates/plots per genotype, giving 60 plots in total. Each plot was 6 m^2^ with a 3 m length and a 2 m width. The space between the plots was 0.2 m. Each plot had 15 rows, and the distance between rows was 0.2 m. There was a buffer zone of an approximately 3 m width around the experimental area.

All the seeds were sown at a depth of 4–6 cm. There was no irrigation during the growing season, so the experiment relied completely on precipitation. Before sowing, compound fertilizer (Naweigao Fertilizer Company, Zhengzhou, China, N-P-K = 25-13-7) was applied to the field at the rate of 750 kg per hectare. All the fertilizer reached the soil plough layer when applied. When the wheat was mature in May, we selected 1 m × 2 m areas in the center of each plot as the sample plots to measure the yield. All the grains in the sample plots were harvested by hand. The yield components, the thousand kernel weight (TKW) and the number of grains per spike (grain number) were recorded at harvest. The number of spikes per m^2^ (spike density) was determined after counting the number of spikes for 2 lines in the center of the sample plots during the filling stage. All the grains produced in the plots were harvested, weighed and stored in mesh bags with labels.

### 2.2. Measurements of the Leaf and Root Anatomical Traits

**Figure 3:**
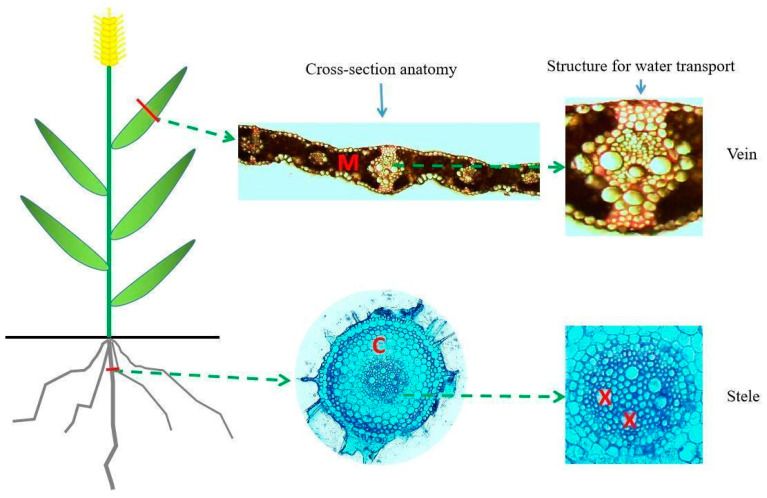
The image on the left illustrates the positions of the samples collected. Leaf segments were excised from the middle part of the flag leaves, and root samples were excised from the seminal root 2 cm below the seed. The images in the middle show the cross-sectional structures of the leaves and roots. The images on the right show structures for water and nutrient transport in leaves (vein) and roots (stele). M refers to leaf mesophyll tissue, C refers to root cortical tissue and X refers to root xylem vessels.

In this experiment, three plants per plot were dug up near the center of each plot, but away from the area of the plot selected for the population yield at the final harvest, to assess their anatomical traits. Then, 1 cm leaf segments were excised from the middle part of the flag leaves, and 1 cm root samples were excised from the seminal root 2 cm below the seeds from which they originated at anthesis (Figure 3). Seminal roots have been widely used in wheat to assess its water transport and/or water use efficiency [28,29,30,31,32]. The collected seminal root sections were critical nodes linking the roots with the stems, which are responsible for transporting water axially from the root xylem to the stem vascular bundle systems at all stages, affecting overall hydraulic conductance and water usage. In each plot, we collected 3 individuals at anthesis, so each genotype had 9 replicates. All the excised samples were fixed in FAA solution (95% ethanol:formaldehyde:glacial acetic acid:distilled water = 18:1:1:5.4) for at least 48 h. Then, the samples were embedded into agarose solvent (6% with distilled water) within 7 mm × 7 mm × 5 mm molds.

Cross-sections (20 μm thick leaf samples and 50 μm thick root samples) were cut using a vibratome (VT1000 S, Leica, Germany) and stored in a centrifuge tube with distilled water in a refrigerator. Then, the cross-sections were stained with 1% safranine solvent, dehydrated in 30%, 50%, 75% and 83% ethanol in sequence, stained with 0.5% Fast Green with 95% ethanol and dehydrated in 95% and then 100% ethanol for 10 s. They were then mounted onto a glass slide with neutral resin (BL704A, Biosharp, Hefei, China) and covered with a cover glass.

The cross-sections were observed and documented under upright light microscopy (Olympus BX35, Tokyo, Japan) at a resolution of 1360 × 1024. The whole root cross-sectional area and leaf vein density were measured at a magnification of 40. All the other parameters were measured at a magnification of 100. Photographic images of the leaf cross-sections were used to measure the leaf mesophyll area, leaf vein area and leaf thickness. Meanwhile, the root cross-sections were used to measure the whole root area, cortex area, stele area, xylem area, etc. Measurements were taken from the photographs using ImageJ software (1.8.0). The definitions and abbreviations for the roots and their anatomical traits are summarized in Table 2.

### 2.3. Statistical Analyses

Regression analyses were used to examine the relationships between the grain yield, yield components and root and leaf anatomical traits. The regression analyses and principal component analysis were performed using R software v4.0.2, and the path analyses were performed using IBM SPSS AMOS 21. ANOVA were performed using GenStat (23rd Edition). Graphs of the daily temperature and precipitation per 10 days were plotted using SigmaPlot v. 14.

## 3. Results

### 3.1. Yield and Yield Components

There were clear differences in the population yield and yield components among the 20 genotypes (*p* < 0.05). The highest yield was 799.07 g m^−2^ from Lunxuan99, and the lowest was 569.45 g m^−2^ from Yunhan20410 (Table S1). The thousand kernel weight (TKW) ranged from 36.05 g to 49.29 g, the number of grains per spike (grain number, GN) ranged from 28.99 to 42.34 and the number of spikes per m^2^ (spike density, SD) ranged from 384.33 to 551.67 (Supplementary Materials Table S1). In the path analysis of the yield components, grain number, TKW and spike density all had positively significant contributions to yield production (*p* < 0.001). Spike density made the greatest contribution among all three yield components to the population yield (Table S2). Grain number made a greater contribution to yield than did TKW.

### 3.2. The Relationships between the Root and Leaf Anatomical Traits and Population Yield

**Figure 4:**
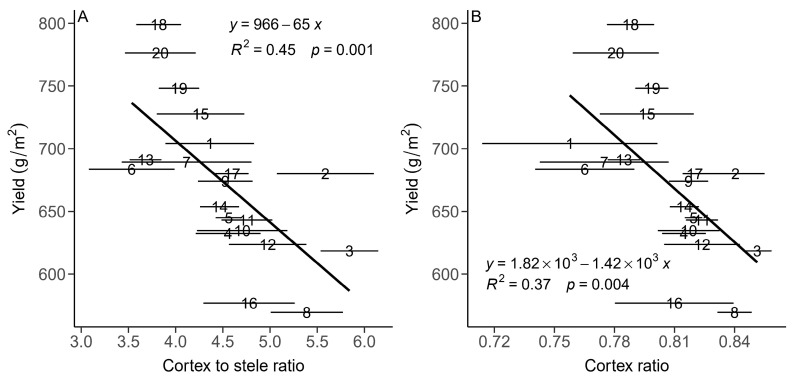
Relationships between means of yield and cortex-to-stele ratio (**A**) and cortex ratio (**B**) for 20 genotypes of winter wheat in central China. A solid line indicates a significant linear relationship. The numbers from 1 to 20 represent the different genotypes. Horizontal error bars (standard error) are shown.

**Figure 5:**
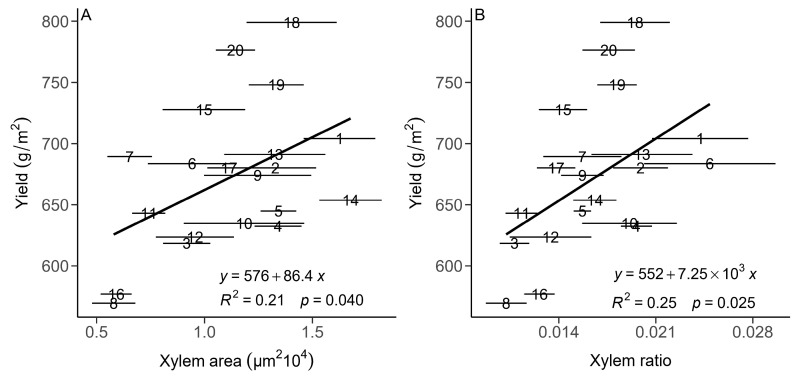
Relationships between mean of yield and xylem area (XR, (**A**)) and xylem ratio (XR, (**B**)) for 20 genotypes of winter wheat in central China. Solid lines indicate significant relationships. The numbers from 1 to 20 represent the different genotypes. Horizontal error bars (standard error) are shown.

There were no significant relationships between yield and root diameter (Figure 4A) or absolute cortex area (CA; Figure 4B). There were significantly negative relationships between yield and the cortex-to-stele ratio (*p* < 0.01, Figure 4A) and yield and the cortex-to-whole-root ratio (cortex ratio, CR; *p* < 0.01, Figure 4B). There were significant positive relationships between the absolute xylem area, relative xylem area (xylem ratio), relative stele area (stele ratio, SR) and yield (*p* < 0.05, Figure 5). For the leaf anatomical traits, there were no clear relationships between yield and leaf thickness (LT), relative leaf mesophyll area (leaf mesophyll ratio, MR; *p >* 0.05) or relative leaf vein area (leaf vein ratio, VR).

### 3.3. Relationships between the Root and Leaf Anatomical Traits and Yield Components

**Figure 6:**
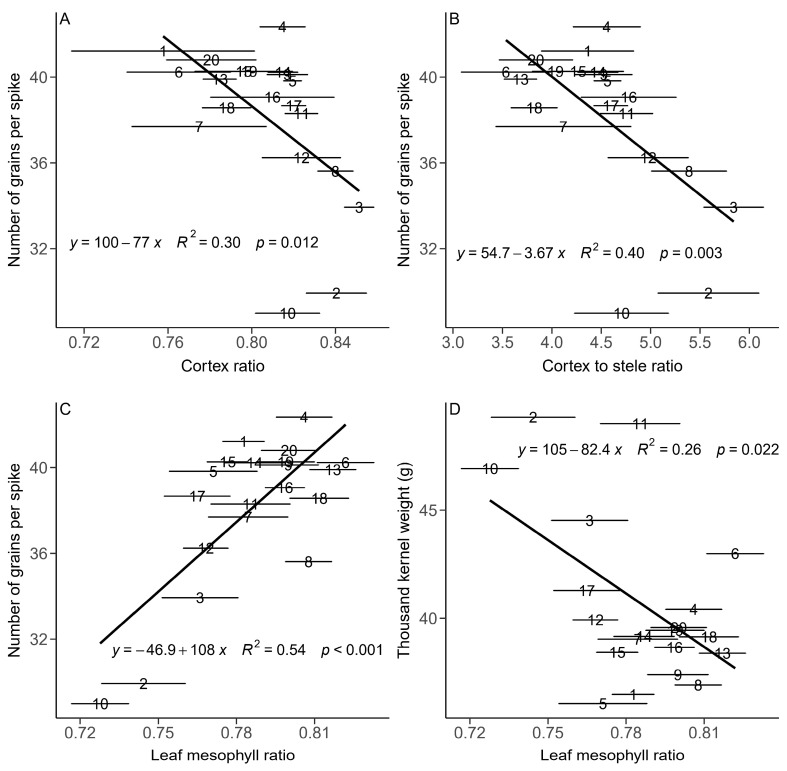
Relationships between means of number of grains per spike and cortex ratio (**A**), cortex-to-stele ratio (**B**) and leaf mesophyll ratio (**C**) and between thousand kernel weight and leaf mesophyll ratio (**D**) for 20 genotypes of winter wheat in central China. A solid line indicates a significant relationship. The numbers from 1 to 20 represent the different genotypes. Horizontal error bars (standard error) are shown.

For the yield components, grain number was negatively correlated with the cortex-to-stele ratio and the cortex ratio (*p* < 0.05, Figure 6) and positively correlated with the stele ratio. However, there were no significant relationships between TKW or spike density and these root anatomical traits (*p >* 0.05). Grain number was positively correlated with the leaf mesophyll ratio (*p* < 0.05, Figure 6) and negatively correlated with the leaf vein ratio. TKW was positively correlated with the leaf vein ratio and negatively correlated with the leaf mesophyll ratio (*p* < 0.05, Figure 6). There was a trade-off between grain number and TKW (Figure 6C,D). There were no significant relationships between spike density and any other anatomical trait.

### 3.4. Results of Principal Component Analysis of Root and Leaf Anatomical Traits

**Figure 7:**
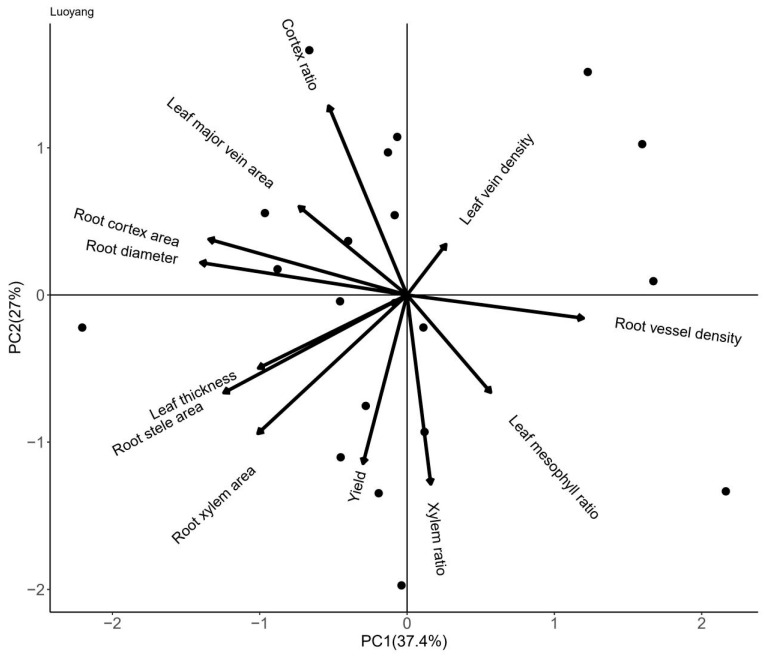
Principal component analysis of root and leaf anatomical traits and yield among 20 genotypes of wheat in central China.

The first principal component mainly reflected root traits relevant to carbon allocation, including root diameter, root cortex area and root cortex ratio (Table 3). The second principal component mainly reflected the yield, leaf thickness and plant traits relevant to water transport, including the root stele area and the root xylem area. Yield was positively correlated with the xylem area and xylem ratio but was not correlated with the cortex area, root diameter or vessel density. The root cortex area was positively correlated with the root diameter but negatively correlated with the root vessel diameter. The leaf mesophyll ratio was negatively correlated with the leaf major vein area (Figure 7).

## 4. Discussion

### 4.1. Root Anatomical Traits and Yield

Our results show significant relationships between several root anatomical traits and winter wheat yield in Luoyang in one year. Although there was no significant relationship between yield and absolute cortex area (CA), there were significant negative relationships between the relative cortex area (cortex ratio; CR), the ratio of the cortex to the stele (CSR) and the population yield. Our results suggest that reduced cortical tissue with potentially reduced root respiration, construction and maintenance costs may be beneficial in terms of yield. This is consistent with the proposed “root ideotype” of “cheaper, steeper and deeper” roots [15,16] for greater exploration of water and nutrients by the wheat population under rainfed conditions [14,33].

Furthermore, reduced root cortical tissue may decrease resistance to radial water transport and increase the efficiency of water uptake from the soil [6]. This result is consistent with previous findings that a reduced root cortical area and reduced cortical cell files and increased root cortical aerenchyma, replacing some living cortical tissue with air, improved maize (*Zea mays* L.) drought tolerance and yield under low-phosphorus and water stress environments [7,8,11]. Our results suggest that reduced cortical costs can improve the crop yield under rainfed conditions. The reproductive organs and roots are competing sinks for photosynthate, so reducing the living root cortical tissue can be beneficial for reproductive growth by increasing carbohydrate availability for reproductive allocation [11].

Our results fit crop evolutionary theory, which predicts a trade-off between individual fitness and the population yield among productive genotypes [17]. Reduced individual fitness with a relatively reduced carbon cortical burden in root systems potentially weakens the competitiveness of the roots, facilitating soil exploration, water acquisition and reproductive allocation on the part of the population, resulting in high yields [34].

**Figure 8:**
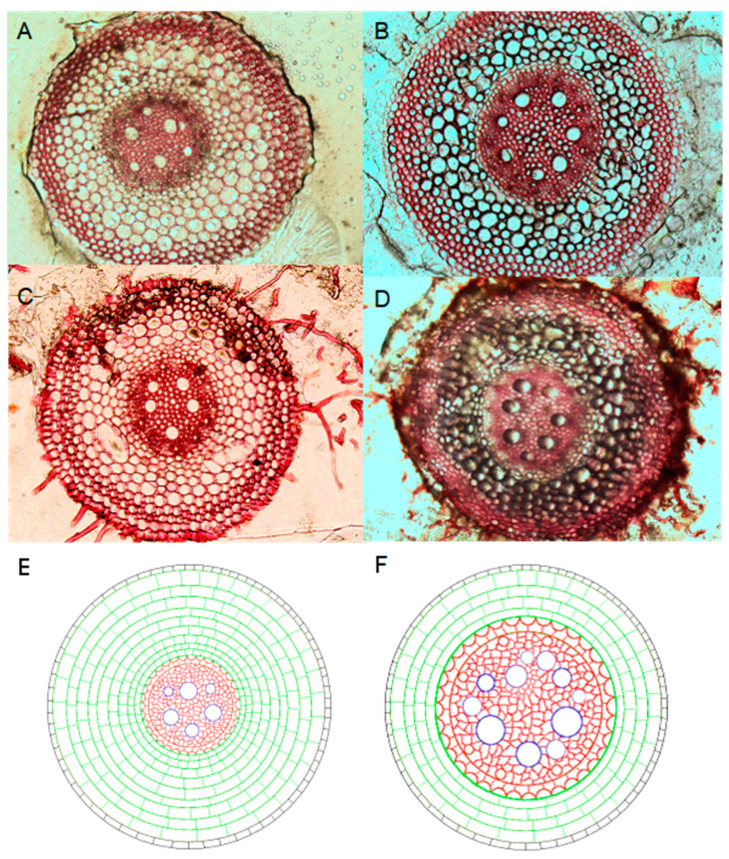
Root anatomy of two low-yield ((**A**) Luohan2 and (**C**) Zhengmai379) and two high-yield genotypes ((**B**) Bainong207 and (**D**) Zhoumai32). A schematic representation of the root anatomy of the low-yield (**E**) and high-yield (**F**) genotypes.

Contrary to our hypothesis, the results showed positive relationships between the absolute xylem area (XA), relative xylem area (xylem ratio; XR), relative stele area (stele ratio; SR) and population yield in this region, suggesting that an increased water transport capacity in the seminal roots could contribute to yield. This contradicts the conclusion of a recent study in which wheat genotypes with low axial conductance conserved water during grain filling under drought conditions, increasing in their grain size and yield [18]. Our results suggest that that genotypes with a larger xylem area at anthesis had a higher axial hydraulic conductance and a higher water transport efficiency, which supports the assumption by Donald (1968) [35] that high-yield phenotypes should have a low biomass and therefore a low competitive ability but high efficiency in the use of environmental resources—in this case, water [34,36,37]. The result is also consistent with a mesocosm study showing that high-yield soybean lines showed an increased metaxylem number, improving the roots’ hydraulic conductivity and water transport [38]. Similarly, a recent study on rice showed that increased water transport efficiency in the roots contributed to increased drought tolerance and high yields [39], maintaining photosynthetic processes in the leaves by supplying sufficient water upward under rainfed conditions [40]. In this study, the genotypes Bainong207 and Zhoumai32 with a high drought tolerance and disease tolerance also showed high yields and large xylem areas (Figure 8). Root anatomy in winter wheat is likely to contribute to the trade-off between carbon cortical costs and water uptake in this region. Thus, a high water transport efficiency with reduced cortical costs and increased xylem input in the roots is likely to play a role in both radial and axial water transport, crop reproductive allocation, drought and disease tolerance and the population yield. In addition to root anatomy, there are other factors that influence the root transport efficiency, e.g., the electrical capacitance in the lateral roots. Future studies should investigate these.

### 4.2. Leaf Anatomical Traits and Yield

Flag leaf thickness was positively correlated with yield. Leaf thickness is an important trait in crops and affects photosynthesis and source–sink efficiency [41]. Our results are consistent with theories addressing rice architecture, which indicate that thick, short and erect flag leaves could promote rice yields [42]. Parallel veins mean that an increase in leaf thickness will occur via an increase in the mesophyll, supplying more carbon for assimilation. Contrary to our expectations, there was no significant relationship between yield and relative leaf mesophyll area ratio. Some high-yield genotypes had a relatively high leaf mesophyll ratio, however, suggesting that for some winter wheat genotypes, more photosynthate being produced by the leaves’ mesophyll cells is conducive to high yields. Although this wheat ideotype has few and small erect leaves with a low competitive ability, it has a high light use efficiency [35,43] with more mesophyll tissue per unit of cross-sectional leaf area.

Yield was not significantly correlated with relative flag leaf vein area, suggesting that high-yield genotypes do not enhance the water transport capacity of their roots with an increased leaf vein area but have a conservative water use strategy, with tight regulation of the water loss from the leaves. Some genotypes with high yields had a relatively low leaf vein ratio. It may be that a lower vein investment with a lower construction cost and potentially low water use by the leaves is beneficial for optimizing the water and carbon use efficiency of whole plants, thereby increasing their yield [25].

The results support our hypothesis that high-yield genotypes can maximize carbon gain by having an increased amount of mesophyll tissue per unit of leaf area while reducing transpirational water loss with a reduced vein area. Stomata are critical sites for carbon dioxide and water vapor exchange, and there is evidence of a positive correlation between stomatal density and yield in dryland wheat in one study (P. Du, unpublished). More studies are needed to investigate how the leaf veins and stomata have evolved together to regulate carbon and water uptake in crops to achieve high yields.

### 4.3. Root and Leaf Anatomical Traits and Yield Components

Yield can be increased via one or more of a plant’s components [44,45]. The path analysis showed that all three yield components (kernel weight, number of grains per spike and spike density) made positive contributions to the population yield, and the number of grains per spike was more important to yield than grain weight (Supplementary Materials). Our results are consistent with the well-established generalization that increases in grain yield occur via increases in grain number rather than increases in grain weight [46].

In our study, the number of grains per spike was negatively correlated with relative cortex area and the cortex-to-stele ratio but positively correlated with relative stele area (stele ratio). This indicates that reduced root cortical tissue and an increased stele investment could promote an increased number of grains per spike via an increased spike length, contributing to the population yield in this region.

Although yield was not correlated significantly with the leaf mesophyll ratio or leaf vein ratio, our results showed that the number of grains per spike was positively correlated with the leaf mesophyll ratio and negatively correlated with the leaf vein ratio. Grain weight was positively correlated with the leaf vein ratio and negatively correlated with the leaf mesophyll ratio, however. The anatomical traits that were correlated with grain weight and grain number per spike were different. This is consistent with the well-established trade-off between grain weight and grain number [47,48]. The leaf mesophyll and leaf vein ratios influenced this trade-off. Reduced relative root cortex and leaf vein areas and increased relative root stele and leaf mesophyll areas are associated with increased grain numbers and yields.

## 5. Conclusions

The 20 genotypes of winter wheat growing in the major wheat-producing area of China showed significant relationships between some anatomical traits and yield. Our results indicate that a high yield was associated with a reduced relative root cortex area and increased absolute and relative root xylem areas. Reduced root cortical tissue may decrease resistance to radial water transport and increase the efficiency of water uptake from the soil. A high yield was associated with reduced carbon consumption and an improved water transport capacity in the roots but not with changes in the regulation of water loss from the leaves. Focus on anatomical traits could contribute to future increases in crop yield.

## Figures and Tables

**Table 1:** Name, year of release, phenology and source of all 20 cultivars of winter wheat used in the experiments.

No.	Cultivar	Year of Release	Days to Flower	Days to Mature	Source
1	Yumai49	2000	183	219	Xiangyun Agricultural Technology Station
2	Zhengmai9023	2002	184	217	Henan Academy of Agricultural Science
3	Luohan2	2003	184	222	Luoyang Agricultural Science Research Institute
4	Zhoumai18	2005	184	221	Zhoukou Academy of Agricultural Science
5	BainongAK58	2005	180	217	Henan Institute of Science and Technology
6	Jimai22	2007	184	220	Shandong Academy of Agricultural Science
7	Luohan12	2008	184	220	Luoyang Agricultural Science Research Institute
8	Yunhan20410	2008	185	219	Shanxi Academy of Agricultural Science
9	Luomai9	2008	185	220	Luohe Academy of Agricultural Science
10	Shannong20	2010	183	219	Shandong Agricultural University
11	Zhongmai175	2011	182	219	Chinese Academy of agricultural science
12	Zhoumai26	2012	182	220	Zhoukou Agricultural University
13	Henong7106	2012	183	222	Hebei Agricultural University
14	Zhengmai101	2013	183	220	Henan Academy of Agricultural Science
15	Bainong207	2013	184	221	Henan Bainong Seed Industry Co. Ltd.
16	Yumai158	2014	185	221	Luohe Academy of Agricultural Science
17	Zhengmai379	2016	184	221	Henan Academy of Agricultural Science
18	Lunxuan99	2016	182	219	Chinese Academy of agricultural science
19	Zhoumai32	2018	183	219	Zhoukou Academy of Agricultural Science
20	Tongmai6	2019	183	221	Center of Tongchuan Agricultural Technology Development

**Table 2:** Definition and abbreviations for the anatomical traits.

Anatomical Traits	Units	Descriptions
Root anatomical traits		
Root diameter	μm	The whole root’s cross-sectional diameter
Cortex area	μm^2^	The area between the exodermis and endodermis
Xylem vessel area	μm^2^	Also named the xylem area, total vessel area
Xylem ratio	%	Root xylem vessel area/total root cross-sectional area ratio
Cortex-to-stele ratio		Root cortex area/total stele area ratio
Cortex ratio		Root cortex area/total cross-sectional area ratio
Stele ratio		Root stele area/total cross-sectional area ratio
Root vessel density		Root vessel number/root stele area ratio
Leaf anatomical traits		
Vein density	mm^−1^	Total number of veins per mm, also known as “vein length per leaf area”
Major vein area	μm^2^	Includes leaf vascular bundle sheath, mechanical tissue, xylem and phloem
Leaf thickness	μm	The average leaf thickness at three positions
Leaf vein area ratio		The total leaf vein area to the total leaf cross-sectional area
Leaf mesophyll area ratio		The total leaf mesophyll area to the total leaf cross-sectional area

**Table 3:** Principal component analysis based on root and leaf anatomical traits in 20 winter wheat genotypes.

Yield and Anatomical Traits	PC1	PC2
Yield	−0.42	0.67
Root anatomical traits		
Root diameter	0.75	0.58
Cortex area	0.80	0.48
Stele area	0.25	0.90
Xylem area	0.01	0.92
Root vessel density	−0.62	−0.51
Cortex ratio	0.86	−0.33
Xylem ratio	−0.69	0.51
Leaf anatomical traits		
Leaf vein density	0.04	−0.28
Major vein area	0.63	0.08
Leaf thickness	0.22	0.71
Leaf mesophyll area ratio	−0.58	0.03

## Data Availability

The data associated with this study have been deposited on Figshare: https://doi.org/10.6084/m9.figshare.24265216 (accessed on 7 October 2023).

## Supplementary Materials

The following supporting information can be downloaded at: https://www.mdpi.com/article/10.3390/plants13081075/s1. Yield and yield components of 20 winter wheat genotypes and a path analysis of yield components. Table S1: Mean yield and yield components (thousand kernel weight, number of grains per spike, and the number of spikes per m^2^ [spike density]) of 20 winter wheat genotypes in the growing season 2020–2021 in central China. Standard error in parentheses; Table S2: The results of path analysis of yield components in winter wheat. The three yield components all had a positively significant influence on yield (*p* < 0.001). Spike density had the highest coefficient among the three yield components. Grain number made a greater contribution to yield than did grain weight; Figure S1: Relationship between yield and year of release. When more than one cultivar was released in the same year, all are included in the average; Figure S2: Relationships between yield and abaxial stomatal density in wheat in a field study in Zhuanglang, Gansu, China (P. Du, unpublished).
