# Supplementary material for: Reduced Root Cortical Tissue with an Increased Root Xylem Investment Is Associated with High Wheat Yields in Central China
Source: Plants (Basel). 2024 Apr 11;13(8):1075. doi: 10.3390/plants13081075 (PMC11054696; doi:10.3390/plants13081075)
Supplement: Supplementary file 1 [file plants-13-01075-s001.zip › plants-2930872-supplementary.pdf]

Supplementary materials

Table S1. Mean yield and yield components (thousand kernel weight, number of grains per spike, and the number of spikes per m^2 [spike density]) of 20 winter wheat genotypes in the growing season 2020-2021 in central China. Standard error in parentheses.

No.	Yield (g)	Thousand kernel weight (g)	Number of grains per spike	Spike density
Tongmai6	704.17 (20.56)	36.48 (1.03)	41.22 (2.33)	480.33 (8.82)
Shannong20	680.09 (46.11)	49.29 (0.97)	29.93 (0.57)	501.67 (33.29)
Luohan2	618.52 (29.73)	44.52 (1.94)	33.93 (0.92)	475.00 (2.08)
Yunhan20410	632.41 (32.47)	40.41 (0.30)	42.34 (1.98)	397.33 (17.38)
Zhoumai18	644.91 (43.15)	36.05 (1.10)	39.82 (0.33)	475.33 (11.46)
Zhongmai175	683.61 (25.90)	42.99 (0.61)	40.23 (0.88)	447.67 (17.80)
Jimai22	689.35 (22.93)	39.03 (1.93)	37.69 (0.67)	522.67 (19.94)
Bainongak58	569.45 (36.15)	36.91 (1.06)	35.61 (1.40)	463.67 (30.47)
Lunxuan99	674.07 (30.07)	37.39 (0.60)	40.12 (0.50)	473.00 (22.05)
Yumai49	634.72 (53.55)	46.92 (1.49)	28.99 (0.59)	506.67 (18.05)
Yumai158	643.05 (41.53)	48.99 (0.71)	38.30 (0.73)	384.33 (17.27)
Luohan12	623.61 (54.26)	39.93 (1.66)	36.24 (1.85)	469.33 (8.45)
Henong7106	691.20 (22.14)	38.38 (0.28)	39.90 (0.21)	469.67 (5.70)
Zhoumai26	653.70 (41.33)	39.16 (0.99)	40.23 (0.60)	443.33 (11.14)
Zhengmai101	727.78 (51.89)	38.43 (0.47)	40.27 (1.70)	478.67 (30.91)
Zhengmai379	576.85 (19.25)	38.65 (0.32)	39.07 (0.89)	413.67 (15.17)
Zhengmai9023	680.09 (51.20)	41.28 (0.82)	38.67 (1.41)	454.00 (22.74)
Bainong207	799.07 (5.22)	39.14 (0.82)	38.57 (1.93)	551.67 (27.94)
Luomai9	748.15 (26.47)	39.44 (0.76)	40.27 (0.54)	503.33 (20.58)
Zhoumai32	776.39 (38.35)	39.57 (1.18)	40.80 (0.35)	509.33 (16.84)
P value	$P < 0.05$	$P < 0.05$	$P < 0.05$	$P < 0.05$

Table S2. The results of path analysis of yield components in winter wheat. The three yield components all had a positively significant influence on yield (p<0.001). Spike density had the highest coefficient among the three yield components. Grain number made a greater contribution to yield than did grain weight.

	**Standardized path coefficient**	**Significance**
Spike density	0.641	P<0.001
Grain number	0.599	P<0.001
Grain weight (TKW)	0.401	P<0.001

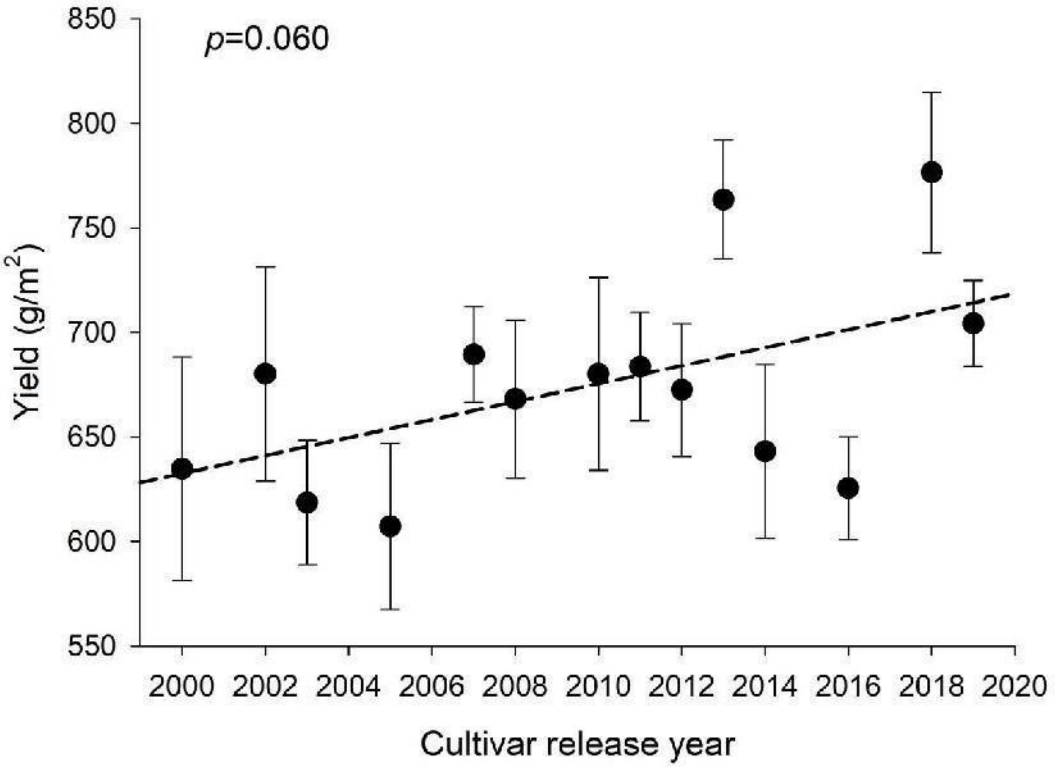

Figure S1. Relationship between yield and year of release. When more than one cultivar was released in the same year, all are included in the average.

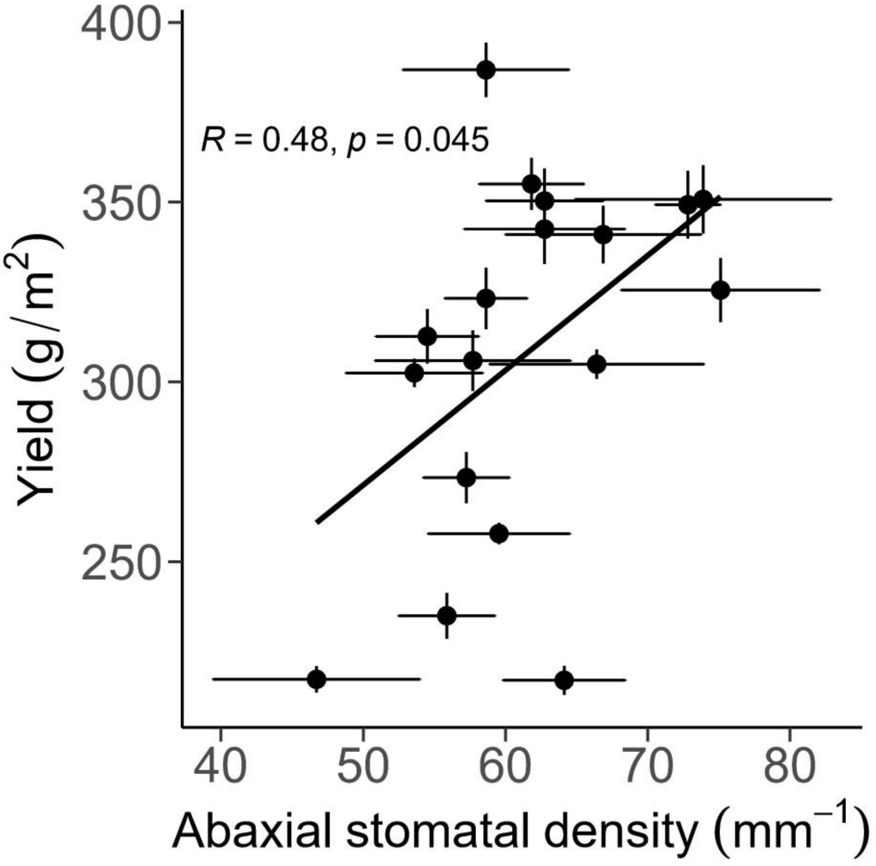

Figure S2. Relationships between yield and abaxial stomatal density in wheat in a field study in Zhuanglang, Gansu, China (P. Du, unpublished).
